# Plasma Proteomic Profiling in Hereditary Breast Cancer Reveals a BRCA1-Specific Signature: Diagnostic and Functional Implications

**DOI:** 10.1371/journal.pone.0129762

**Published:** 2015-06-10

**Authors:** Domenica Scumaci, Laura Tammè, Claudia Vincenza Fiumara, Giusi Pappaianni, Antonio Concolino, Emanuela Leone, Maria Concetta Faniello, Barbara Quaresima, Enrico Ricevuto, Francesco Saverio Costanzo, Giovanni Cuda

**Affiliations:** 1 Dpt. of Experimental and Clinical Medicine, Magna Græcia University of Catanzaro, Salvatore Venuta University Campus, Catanzaro, Italy; 2 Medical Oncology, S. Salvatore Hospital, University of L'Aquila, L'Aquila, Italy; University of Salerno, Faculty of Medicine and Surgery, ITALY

## Abstract

**Background:**

Breast cancer (BC) is a leading cause of death among women. Among the major risk factors, an important role is played by familial history of BC. Germ-line mutations in BRCA1/2 genes account for most of the hereditary breast and/or ovarian cancers. Gene expression profiling studies have disclosed specific molecular signatures for BRCA1/2-related breast tumors as compared to sporadic cases, which might help diagnosis and clinical follow-up. Even though, a clear hallmark of BRCA1/2-positive BC is still lacking. Many diseases are correlated with quantitative changes of proteins in body fluids. Plasma potentially carries important information whose knowledge could help to improve early disease detection, prognosis, and response to therapeutic treatments. The aim of this study was to develop a comprehensive approach finalized to improve the recovery of specific biomarkers from plasma samples of subjects affected by hereditary BC.

**Methods:**

To perform this analysis, we used samples from patients belonging to highly homogeneous population previously reported. Depletion of high abundant plasma proteins, 2D gel analysis, liquid chromatography-tandem mass spectrometry (LC-MS/MS) and bioinformatics analysis were used into an integrated approach to investigate tumor-specific changes in the plasma proteome of BC patients and healthy family members sharing the same BRCA1 gene founder mutation (5083del19), previously reported by our group, with the aim to identify specific signatures.

**Results:**

The comparative analysis of the experimental results led to the identification of gelsolin as the most promising biomarker.

**Conclusions:**

Further analyses, performed using a panel of breast cancer cell lines, allowed us to further elucidate the signaling network that might modulate the expression of gelsolin in breast cancer.

## Introduction

Breast cancer (BC) is the most commonly diagnosed cancer in women worldwide, representing about 12% of all new cancer cases and 25% of all cancer cases in women [1]. Due to the high morphological and genetic heterogeneity, traditional methods for subgrouping BC, which rely on pathological and clinical data can only partially reflect the clinical variety of the disease. Molecular profiling has been shown to be well suited to phenotypic characterization of BC and to discover potentially new molecular classes among cancers with similar histological appearance [2].

It is estimated that 5%–10% of all breast and ovarian cancer (BOC) cases are genetically inherited, and the BC susceptibility genes BRCA1 and BRCA2 have been identified as being responsible for 21%–40% of these cases [3].

Women who carry a germline mutation in BRCA1 have a lifetime risk of 50%–85% of developing breast cancer and 12%–60% of developing ovarian cancer. BRCA1-mutated breast tumours are generally ER, PgR, and HER2/neu negative and poorly differentiated with a poor prognosis [4].

The BRCA1 tumour suppressor gene encodes for a multifunctional protein that has been implicated in many normal cellular functions such as DNA repair, transcriptional regulation, cell-cycle checkpoint control, and ubiquitination [5, 6].

A cell carrying a mutant BRCA1 gene, which therefore lacks functional BRCA1 protein, shows a decreased ability to repair damaged DNA. In animal models, this defect may cause genomic instability [7]. In humans, BRCA1-positive breast tumours are characterized by a large number of chromosomal changes, some of which differ depending on the genotype [8].

Early diagnosis of BC is difficult due to a lack of specific symptoms and to a limited understanding of breast tumorigenesis.

Presently, the diagnosis of BC relies on an integrated approach using clinical and physical examination, imaging mammography and ultrasound, as well as histopathology. Although plasma biomarkers have not yet displayed a major role in breast cancer diagnostic or prognostic practice, an effective biomarker panel in an easily accessible biological fluid would be a valuable and minimally invasive tool [9–11].Therefore, the analysis of plasma proteome in BC patients might be an important step to achieve more accurate, sensitive and specific diagnostic/prognostic standards [12].

However, the identification and characterization of disease-related plasma biomarkers is quite challenging because of the heavy presence of proteins such as albumin, immunoglobulins, transferrin, lipoproteins, which constitute ~ 90% of the protein content of serum. These high abundance proteins can interfere with proteomics investigation of less-represented signalling proteins. Therefore, the reduction of sample complexity is considered an essential step in the analysis of the plasma proteome [13, 14].

To this end, our group has used a robust and high-throughput quantitative method with sensitivity and high-resolving power. This method is based on an integrated proteomic approach which includes: selective removal of the most abundant plasma proteins, 2D gel electrophoresis and LC-MS/MS analysis, followed by identification of the main networks in which deregulated proteins are involved and validation of results through western blot analysis.

In this study, we have performed a molecular profiling of plasma proteome from individuals (BC-affected and non-affected carriers) bearing a BRCA1 germline mutation in their genome. More specifically, we focused on families of Calabrian origin, with hereditary BOC, due to a frameshift mutation in one copy of the BRCA1 gene and leading to a stop codon at position 1670 (5083del19) of the BRCA1 [15]. This mutation, which appears to be highly prevalent in our region, allowed us to perform a reliable proteomic analysis in a highly homogeneous genetic background.

## Materials and Methods

### Patients

The study was approved by the “Comitato Etico Azienda Ospedaliera Universitaria Mater Domini” University Magna Graecia of Catanzaro. Blood samples were obtained from each affected patient and family members after obtaining written consent. Twelve subjects were enrolled for the study.

### Sample collection

Plasma samples were collected according with Plasma Proteome Project guidelines; we collected four plasma samples from patients with inherited BC, bearing a founder mutation on the BRCA1 gene, four plasma samples from healthy family members sharing the same mutation (unaffected carriers) and four healthy relatives, free of BRCA1-gene defects (Table 1). Approximately 4 ml of blood were drawn by venipuncture and collected in K2EDTA tube. The samples were centrifuged within 2 hours of collection at 1.300 x g for 10 minutes, and resulting plasma was aliquoted into silicone tubes and stored at -80°C until use.

**Table 1:** List of analyzed samples.

SUBJECT	BREAST CANCER	BRCA1 FOUNDER MUTATION CARRIER
A1	YES	CARRIER
A2	YES	CARRIER
A3	YES	CARRIER
A4	YES	CARRIER
CN1	NO	CARRIER
CN2	NO	CARRIER
CN3	NO	CARRIER
CN4	NO	CARRIER
NN1	NO	NO CARRIER
NN2	NO	NO CARRIER
NN3	NO	NO CARRIER
NN4	NO	NO CARRIER

### Depletion of high-abundance plasma proteins

Depletion of high abundant proteins was performed using the Multiple Affinity Human-7 (Hu-7) Removal System (Agilent Technologies), an HPLC column that removes the seven most abundant plasma proteins including: albumin, IgG, IgA, transferrin, antitrypsin, haptoglobin and fibrinogen. [16]

Depletion was performed following the manufacturer’s protocols; for each sample, 2mg of plasma proteins were diluted four times in the manufacturer’s Buffer A and filtered through a 0.22 μm spin filters, for removal of particulates. The liquid chromatography separations were conducted on an automated ÄKTA FPLC (GE Healthcare). Briefly, 120 μl of diluted sample was injected onto the MARS column in 100% Buffer A, at a flow rate of 0.25 ml/min for 10 min. After collection of the flow-through fraction, the column was washed with buffer “A” at a flow rate of 1,0 ml/min, and the bound proteins were eluted with 100% Buffer B (a low pH urea buffer) at a flow rate of 1.0 ml/min for 7 min. Afterwards, the column was regenerated by equilibration in 100% Buffer A for 11 min, for a total run cycle of 28 min. The flow-through fractions (containing the low abundance proteins from two sequential injections were collected, pooled and buffer-exchanged into 20 mM Tris–HCl, pH 7.4, the resultant pools were concentrated using spin concentrators with 5 kDa MW cutoff (Agilent Technologies). The low-abundance fractions were either analyzed immediately or aliquoted and stored at -80_C until use.

### 1D Gel electrophoresis

SDS-PAGE analysis of human plasma before and after depletion was performed to evaluate high abundant protein removal. Total protein content of plasma samples was determined using the Bradford Protein Assay (Bio-Rad) according to the manufacturer’s instructions with human serum albumin (Sigma Aldrich) as standards [17]. Briefly, for SDS-PAGE gel experiments 20 μg of each sample were diluted with Laemmli buffer and incubated at 100°C for 5 min. Following incubation all sample were loaded onto a 12% SDS-polyacrylamide gel and run at 50 V [18]. Gels were then stained with EZBlue Gel Staining Reagent, (Sigma Aldrich).

### 2D Gel electrophoresis analysis

Plasma samples, depleted of high abundant proteins, were pooled to create three groups (affected patients, healthy carriers and controls) in the attempt to minimize intra-class sample variability. Resulting pools were subjected to high resolution 2D [19]. For each pool 130 μg of proteins were diluted into Isoelectrofocusing (IEF) sample buffer containing 8 M urea, 4% CHAPS, 0.1 M DTT, 0.8% pH 3–10 nonlinear (NL) carrier ampholyte buffer. IEF was carried out on non-linear immobilized pH gradients (pH 3–10 NL; 24-cm-long IPG strips; GE Healthcare). The first dimension IPG strips were run on a GE Healthcare IPGphor unit, until a total of 70 000 Vh was reached. Prior to SDS-PAGE, IPG strips were equilibrated with a dithiothreitol (10 mg/mL) SDS equilibration solution followed by a treatment with iodoacetamide (25 mg/mL) SDS equilibration solution as described in the GE Healthcare Ettan DIGE protocol. Second dimension separation was run on 10% SDS-polyacrylamide gels, (2W/gel; 25°C) until the bromophenol blue dye front reached the end of the gels [20].Gels were stained with MS-compatible silver staining procedure [21–23]. Each pool analysis was performed in triplicate.

Gel image analysis was carried out using the Image Master 2D-Platinum software, version 6.0 (GE Healthcare). The spot auto-detect function was used for all group comparisons applying identical parameters. Groups were matched automatically and corrected manually if necessary. Differences in protein expression were identified using the relative volume (%Vol) option of the software. This option allows the data to be independent of experimental variations between gels caused by differences in loading or staining [24]. Analysis was performed using three independent experiments, respectively. All data were presented as mean ± SEM (N), where SEM represents the standard error of the mean and N indicates the number of experimental repeats. Unpaired t-test was used to compare protein levels in each data sets compared to control group. A two-sided p-value < 0.05 was considered statistically significant. Data were plotted using Excel spreadsheet (Microsoft).

Electrophoretic spots, obtained from analytic 2D gels, were manually excised, destained, and acetonitrile-dehydrated. They were then rehydrated in trypsin solution, and in-gel protein digestion was performed by overnight incubation at 37°C [25]).

The resulting tryptic peptides were purified by Pierce C18 Spin Columns (Thermo Fisher Scientific Inc.) according to the manufacturer’s procedure, eluted with 40μL of 70% acetonitrile and dehydrated in a vacuum evaporator [26]. Each purified tryptic peptide was analyzed through Nanoscale LC-MS/MS.

### Nanoscale LC-MS/MS analysis

LC-MS/MS analysis was performed using an Easy LC 1000 nanoscale liquid chromatography (nanoLC) system (Thermo Fisher Scientific, Odense, Denmark). The analytical nanoLC column was a pulled fused silica capillary, 75 μm i.d., in-house packed to a length of 10 cm with 3 μm C18 silica particles from Dr. Maisch (Entringen, Germany). The peptide mixtures were loaded at 500 nL/min directly onto the analytical column. A binary gradient was used for peptide elution. Mobile phase A was 0.1% formic acid, 2% acetonitrile, whereas mobile phase B was 0.1% formic acid, 80% acetonitrile. Gradient elution was achieved at 350 nL/min flow rate, and ramped from 0% B to 30% B in 15 minutes, and from 30% B to 100% B in additional 5 minutes; after 5 minutes at 100% B, the column was re-equilibrated at 0% B for 10 minutes before the following injection. MS detection was performed on a quadrupole-orbitrap mass spectrometer Q-Exactive (Thermo Fisher Scientific, Bremen, Germany) operating in positive ion mode, with nanoelectrospray (nESI) potential at 1800 V applied on the column front-end via a tee piece. Data-dependent acquisition was performed by using a top-5 method with resolution (FWHM), AGC target and maximum injection time (ms) for full MS and MS/MS of, respectively, 70,000/17,500, 1e6/5e5, 50/400. Mass window for precursor ion isolation was 2.0 m/z, whereas normalized collision energy was 30. Ion threshold for triggering MS/MS events was 2e4. Dynamic exclusion was 15 s.

Data were processed using Proteome Discoverer 1.3 (Thermo Fisher Scientific, Bremen, Germany), using Sequest as search engine, and the HUMAN-refprot-isoforms.fasta as sequence database. The following search parameters were used: MS tolerance 15 ppm; MS/MS tolerance 0.02 Da; fixed modifications: carbamidomethylation of cysteine; variable modification: oxidation of methionine, phosphorylation of serine, threonine and tyrosine; enzyme trypsin; max. missed cleavages 2; taxonomy Human.

Protein hits based on two successful peptide identifications (Xcorr> 2.0 for doubly charged peptides, >2.5 for triply charged peptides, and >3.0 for peptides having a charge state >3) were considered valid.

### Pathway analysis

Ingenuity Pathway analysis (Ingenuity Systems, www.ingenuity.com) was performed to examine functional correlations within differentially expressed proteins. IPA constructs hypothetical protein interaction clusters on the basis of a regularly updated Ingenuity Pathways Knowledge Base.

Data sets containing protein identifiers and corresponding expression values were uploaded into the application. Proteins differentially expressed were overlaid onto global molecular networks developed from information contained in the knowledge base. Networks were then algorithmically generated based on their connectivity. Networks were “named” on the most common functional group(s) present. Canonical pathway analysis acknowledged function-specific proteins significantly present within the networks [27]. Each analysis was statistically evaluated by the Fischer exact test. This was used to calculate a p-value determining the probability that each biological function and/or disease assigned to that network is due to a random event.

### Western Blotting on plasma samples

Western blot analysis was done to verify the expression of gelsolin in cancer and healthy carrier patients compared to healthy control.

Equal amounts of plasma proteins (50μg/lane) were resolved by 10% SDS-polyacrylamide gel electrophoresis and transferred to nitrocellulose membranes (Bio-Rad).

After addition of the blocking mixture, the membranes were incubated with anti-gelsolin goat monoclonal antibody (clone C-20 Santa-Cruz) at appropriate dilutions at 4°C for 2 h. The signal was detected using anti-goat horseradish peroxidase-conjugate secondary antibodies, and ECL (Santa Cruz).

Serum protein concentration for each sample was measured in triplicate using the dye-binding protein (Bio-Rad) with human serum albumin as standard curve. To ensure uniform gel loading, the membranes were stripped for 30 min at 50 C in 62.5 mm Tris-HCl (pH 6.8), 2% SDS, and 100 mm β-mercaptoethanol, blocked in 2% BSA, and reprobed with specific γ-tubulin horseradish peroxidase-conjugate primary antibodies, and ECL (Santa Cruz) accordingly to Seonyoung C. et al. [28–29].

### Cells culture

MCF-7 and HCC1937 cell lines were purchased from the American Type Culture Collection (Rockville, MD, USA).

HCC1937 cells were grown in RPMI 1640 medium (Life Technologies, Paisley, UK), while MCF-7 were grown in Dulbecco’s modified Eagle’s medium (DMEM) (Life Technologies). All media were supplemented with 10% fetal bovine serum (FBS), 2mM L-glutamine, 100 mg/ml streptomycin and 100U/ml penicillin. All cell lines were cultured at a constant temperature of 371C in a 5% carbon dioxide (CO2) humidified atmosphere.

### EGF treatment

Both MCF-7 and BRCA1 cells lines were treated with EGF to induce the expression of BRCA1; confluent cells (70%) were serum starved for 12 h and then incubated for 3, 6 and 12 h in the presence of EGF (50 ng/ml). After incubation, the cells were collected and lysed as described later [30–31].

### Short interfering RNA

Transient BRCA1 interference was done in MCF-7 cells line for 24h, 48h and 72h.

Transfections were carried out using Lipofectamine 2000 reagent (Invitrogen, Paisley, UK), as outlined in the manufacturer's instructions. Oligo siRNA/Brca1 duplex were obtained from Sigma Aldrich and used at a final concentration of 100nM. [32].

### Western blotting on cell lysates

Cells lines were washed with PBS and lysed at 0°C for 30 min using lysis buffer (120 mM NaCl, 30 mM KCl, 0.1% DTT, 0.5% Triton X-100) supplemented with protease and phosphatase inhibitor cocktail (Halt Protease Inhibitor Cocktail/ Halt Phosphatase Inhibitor Cocktail, Thermo Fisher Scientific Inc.). Cell lysate was sonicated at 4°C for 10 sec and subsequently centrifuged at 15 000x g for 20 min. Supernatant was carefully removed and protein content was measured by the Bradford method (Bio-Rad, Hercules, CA); and the supernatants were stored at 80°C.

Equal amounts of proteins extracts were separated on a 4–15% SDS PAGE precast gel (Bio-Rad), and transferred to a nitrocellulose membrane followed by immunoblotting.

Rabbit monoclonal antibody against BRCA1 (clone D-20, Santa Cruz) was used at a final concentration of 1μg/ml. Goat monoclonal antibody against gelsolin (clone C-20, Santa Cruz) was used at a final concentration of 1μg/ml.

HRP-conjugated γ-Tubulin (clone C-20, Santa Cruz) was used at a final concentration of 1μg/ml to ensure equal amount of protein loading.

The signal was detected using anti-goat horseradish peroxidase-conjugate secondary antibodies, and developed by enhanced chemiluminescence (Santa Cruz)

### Statistical Analysis

Western blot signals were quantified by Quantity One software (Biorad) and data were analyzed and plotted using Excel spreadsheet (Microsoft), and expressed as mean ± SEM (N), where SEM represents the standard error of the mean and N indicates the number of experimental repeats. Unpaired t-test was used to compare protein levels in each data set. A two-sided p-value <0.05 was considered statistically significant.

### Co-immunoprecipitation (Co-IP) assay

For immunoprecipitations, 6x10^10^ of MCF-7 and HCC1937 Cells were harvested and lysed in lysis buffer (20 mM NaCl, 30 mM KCl, 0.1% DTT, 0.5% Triton X-100, supplemented with protease and phosphatase inhibitor cocktail, Halt Protease Inhibitor Cocktail/ Halt Phosphatase Inhibitor Cocktail, Thermo Fisher Scientific Inc.); cell lysates were clarified by centrifugation at 18,000x*g* for 30 min. BRCA1 was immunoprecipitated with 10 ng of anti-BRCA1 monoclonal antibody (clone D-20, Santa Cruz) during an overnight incubation with 1 ml of total cells extract (0.5 μg/μl). 20 μl of resuspended volume of Protein A/G PLUS-Agarose (Santa Cruz) were added to the mixture and incubated at 4° C on rotating device for 2 hour. Immune complexes were collected by low speed centrifugation, washed three times in 1 ml lysis buffer, and boiled in 20 μl of SDS loading buffer; denatured proteins were separated by SDS-polyacrylamide gel electrophoresis (4–15% Bio-Rad precast gel). Proteins were transferred to Nitrocellulose membrane, which was blocked in 5% nonfat milk, 150 mM NaCl, 10 mM Tris (pH 8.0), and 0.05% Tween. Immunoblots were performed with rabbit anti-ATF1 antisera at 1 mg/ml (C-20 Santa Cruz.) and developed by enhanced chemiluminescence (Santa Cruz).

### Chromatin immunoprecipitation (ChIP)

ChIP was performed as previously described [33]. Cells (12x106) were treated with 1% formaldehyde directly into the media for 10 min at room temperature on a rocking platform. The cells were then washed and scraped with phosphate-buffered saline and collected by centrifugation at 700×g for 4 min at 4°C, resuspended in cell lysis buffer (10mM Hepes pH 8.0, 85mM KCl, 0.5% NP-40, protease inhibitor 1 μg/ml, leupetin, 1μg/ml aprotinin, 1mM PMSF) and incubated on ice for 10 min. The pellet was resuspended in lysis buffer (50mM Tris–HCl pH 8.1, 10mM EDTA, 1% SDS, protease inhibitor) and incubated on ice for 10 min. The DNA was sonicated to give fragments of approximately 500 bp. Initially, optimum conditions for sonication were determined by agarose gel analysis after reversing cross-links and precipitated DNA. The lysate was diluted 5-fold in ChIP IP buffer (0.01% SDS, 1% Triton-X-100, 1.2mM EDTA, 16.7mM Tris pH 8.1, 167mM NaCl, protease inhibitor 1μg/ml leupetin, 1μg/ml aprotinin, 1mM PMSF) and precleared with 100μl of protein A beads, which has been pre-adsorbed with sonicated salmon sperm DNA for 90 min at 4°C on a rotary mixer. Beads were collected by centrifugation at 2000×*g*, and chromatin solution was transferred to a fresh microcentrifuge tube. At this stage, the solution was split into microcentrifuge tubes and immunoprecipitated with 5 μg of ATF1 (C41-5.1 Santa Cruz) antibodies overnight at 4°C on a rotating wheel. The immune complexes were then captured with 70μl of protein A beads, prepared as described above, for 3 h at 4°C on a rotating wheel. The beads were then washed with 1ml of ChIP buffer 1 (0.1% SDS, 1% Triton-X-100, 2mM EDTA, 20mMTrispH8.1, 150mMNaCl, PMSF), ChIP buffer 2 (0.1% SDS, 1% Triton-X-100, 2mMEDTA, 20mMTris pH 8.1, 500mM NaCl, PMSF), ChIP buffer 3 (0.25M LiCl,1%NP-40,1%deoxycholate, 1mMEDTA, 10mM Tris pH 8, PMSF, DTT), and finally twice with TE 1X and protease inhibitor 1μg/ml leupeptin, 1μg/ml aprotinin, 1mM PMSF. The protein–DNA complexes were then eluted by adding 250 μl of ChIP elution buffer (1%SDS, 0.1MNaHCO3) to the beads and vortexing before incubating at room temperature for 15 min. After centrifugation, the eluate was transferred to a fresh tube and the elution process was repeated with the beads. The eluates were then combined; the cross-links were reversed by adding 1 mg/ml RNAse, 5mM NaCl and incubating for 4 h at 65°C. After centrifugation, the pellet was resuspended in 100 μl ofH2Oand 2μl of 0.5mM EDTA, 4 μl of 1M Tris pH 6.5 and 1μl of proteinase K (20 mg/ml) were added and incubated for 1 h at 45°C. The DNA was then recovered by phenol–chloroform extraction. DNA purity and concentration both for IP than for Input were determined using a NanoDrop.

### Quantitative real-time PCR (qPCR)

Real-time PCR analysis was performed in a total volume of 25 μl containing 12.5 μl of real-time PCR Master Mix (Exilent SYBR Green, Exiqon), 500 nmole each of forward and reverse primers, and 20ng input, IP, or mock-IP (CK) DNA samples as templates. After incubation at 50°C for 2 minutes and at 95°C for 10 minutes, the mixtures were subjected to 40 amplification cycles (15 seconds at 95°C for denaturation and 1 minute for annealing and extension at 62°C). Incorporation of SYBR Green dye into PCR products was monitored in real time using an IQ5 real-time PCR machine (BIORAD). We obtained a CT value from each amplification curve using the software provided by the manufacturer (Bio-Rad).

The primers for target gelsolin promoter amplification were designed using Primer designing tool (http://www.ncbi.nlm.nih.gov/tools/primer-blast/). According with literature the primers were complementary to the promoter over the region -134 –-3.

The amplifications were performed with the following primers set:

gelsolin promoter forward 5_ CCCATGAAGCGGCAATTCAG 3_

gelsolin promoter reverse 5_ TCGGAGTCAGAAGGCCTGG

Fold differences between samples were calculated as previously described [34]. Briefly, the ΔCt value for each sample was calculated according to the equation: ΔCt [Ct (sample)—Ct (input)]. Next, the ΔΔCt was calculated by ΔΔCt = ΔCt (IP sample)- ΔCt (mock-IP control). Finally, the fold difference between the IP sample and mock-IP control was calculated as 2^(-ΔΔCt)^.

## Results

### Depletion of high-abundance plasma proteins

**Fig 1:**
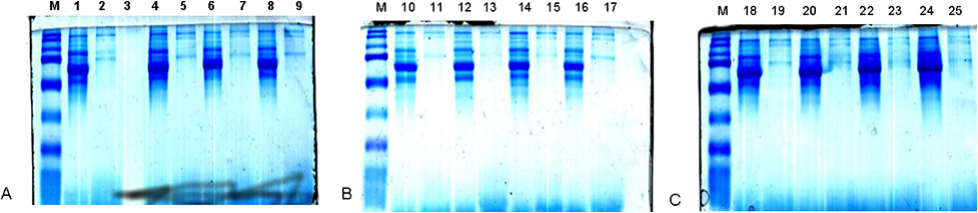
SDS-PAGE gels stained with Coomassie blue, before and after depletion of the high abundant proteins. Lanes 1, 4, 6, 8, 10, 12, 14, 16, 18, 20, 22 and 24 are crude plasma samples from subjects enrolled for the analysis. Lanes 2, 5, 7, 9, 11, 13, 15, 17, 19, 21, 23 and 25 are MARS “Top-7” HPLC-treated plasma samples for depletion of the high abundance proteins. Lane 3 is empty; M: molecular weight standard. 20 μg of proteins were loaded in each lane.

The large dynamic range of protein abundance in plasma represents a substantial analytical challenge. Removal of abundant plasma proteins using antibody capture approaches is a common and attractive mean to reduce sample complexity and to aid the analysis of lower abundance proteins of interest. Here, plasma most abundant proteins (albumin, IgG, antitrypsin, IgA, transferrin, haptoglobin, fibrinogen) were depleted using the MARS “Top-7” HPLC system (Agilent). HPLC depletion chromatogram for each run is shown in supporting S1 Fig. SDS-PAGE was applied to the samples before and after depletion to confirm the columns efficiency. As shown in Fig 1, the most abundant band around the 70 kDa area, which corresponds to albumin, was markedly reduced in the depleted samples. In addition, replicate analysis demonstrated high column reproducibility, linearity and efficient removal of abundant proteins.

### 2D Gel electrophoresis analysis

**Fig 2:**
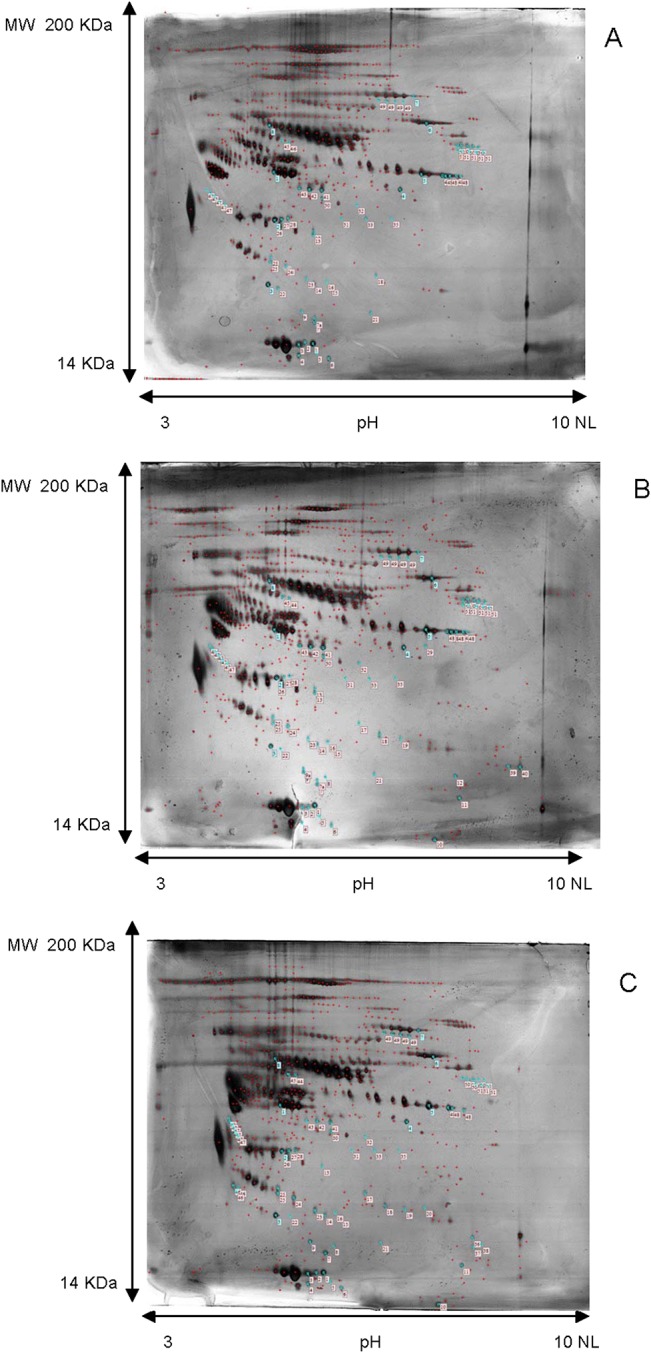
2D gel electrophoresis analysis. Representative 2d gel electrophoresis maps of plasma samples pools for (**A**) patients with breast cancer, carrier of founder mutation, (**B**) Healthy familiar sharing the same mutation and (**C**) Healthy control. Molecular mass separation is 200–10 kDa (top to bottom). Numbered spots indicate proteins that have statistically significant differential expression between groups according to the Image master 2D Platinum 7.0 software (GE Healthcare).

To identify specific biomarkers with diagnostic potential, we compared the 2D gel plasma protein profiles of patients carrying the BRCA1 founder mutation with healthy controls; an additional analysis on plasma obtained from healthy carries (i.e. family relatives bearing the BRCA1 mutation but clinically unaffected) was performed in the attempt to find prognostic markers. The representative 2D maps are shown in Fig 2.

After automatic spot detection, background subtraction and volume normalization, about 629 protein spots were detected in BC patients, 747 in healthy carriers and 700 in healthy controls.

Following the comparative analysis among the 3 groups, 48 spots of interest (matches) were manually excised from the gels, trypsin digested, and used for tandem mass spectrometry analysis. Only reproducibly detected spots were subjected to statistical analysis. A list of up- or down regulated proteins is provided as S1 Table. Thirty of these proteins were present in single spots, while the rest were found in two or more spots. These latter spots might be the result of posttranslational modifications including proteolytic cleavages of specific proteins. Supporting information shows the list of the 48 distinct protein isoforms differentially expressed between the three sample groups and ranked by statistical significance from highest to lowest.

### Pathway analysis

The software IPA (Ingenuity Systems, www.ingenuity.com) was used to evaluate the significant canonical pathways and networks associated to differentially expressed proteins, identified by MS analysis;

We considered only proteins deregulated in a statistically significant way, and then discriminatory between the groups; to this purpose, we included in the analysis only proteins that show a fold change of at least 1.8 (p value<0.05) with respect to control group.

We performed two different analysis: i) for proteins differentially expressed in the plasma samples of affected patients vs controls, we included 33 protein spot identifications (listed in S2 Table), some of which have an identical accession number because are products of post-translational modifications, therefore matching in IPA analysis to 23 unique proteins; ii) for proteins differentially expressed in the plasma samples of healthy carriers vs controls, we included 32 protein spot identifications (listed in S3 Table) which in IPA database matched to 22 unique proteins.

**Fig 3:**
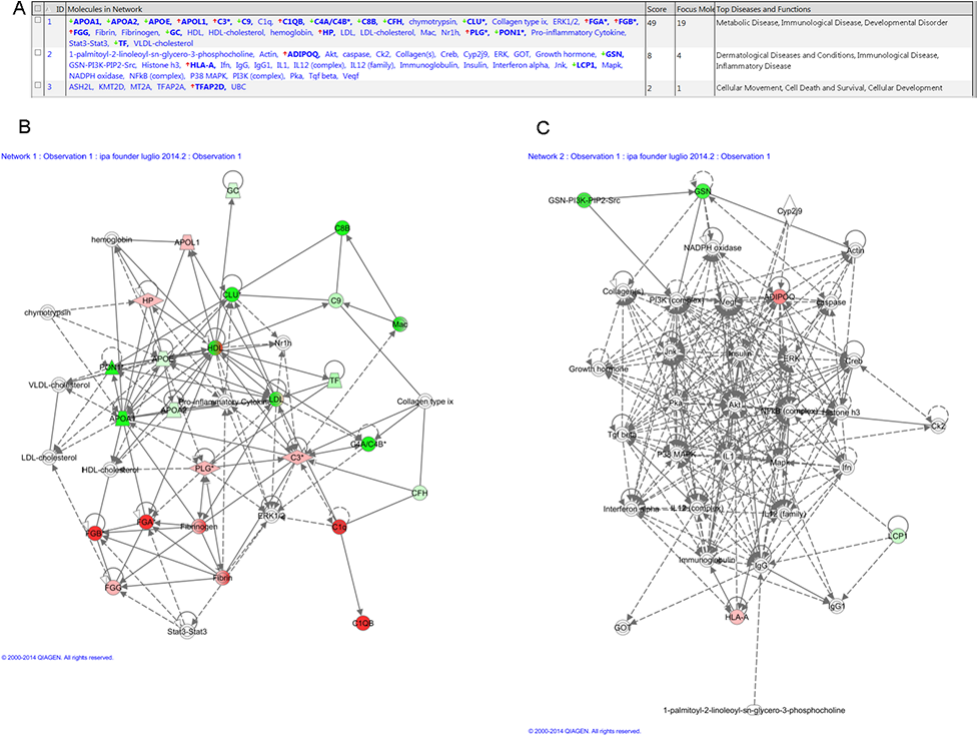
Ingenuity pathway analysis of proteins differentially regulated. (A) List of networks generated from IPA and significantly modulated (log p-value) in cancer patients vs. controls. Top network (B) 1 and (C) 2.

Two domains are covered by the functional pathway analysis: networks and diseases. With regard to the network analysis, we first explored the network characteristics of the proteins differentially expressed in cancer patients vs. healthy controls. IPA analysis predicted four networks (Fig 3A) of interacting protein clusters. The most representative was the first, with a score of 49, in which 19 nodes were enriched and had functions associated with Metabolic Disease, Immunological Disease, Developmental Disorder (Fig 3B). The associated functions of the second network were Dermatological Diseases and Conditions, Immunological Disease, and Inflammatory Disease (Fig 3C).

**Fig 4:**
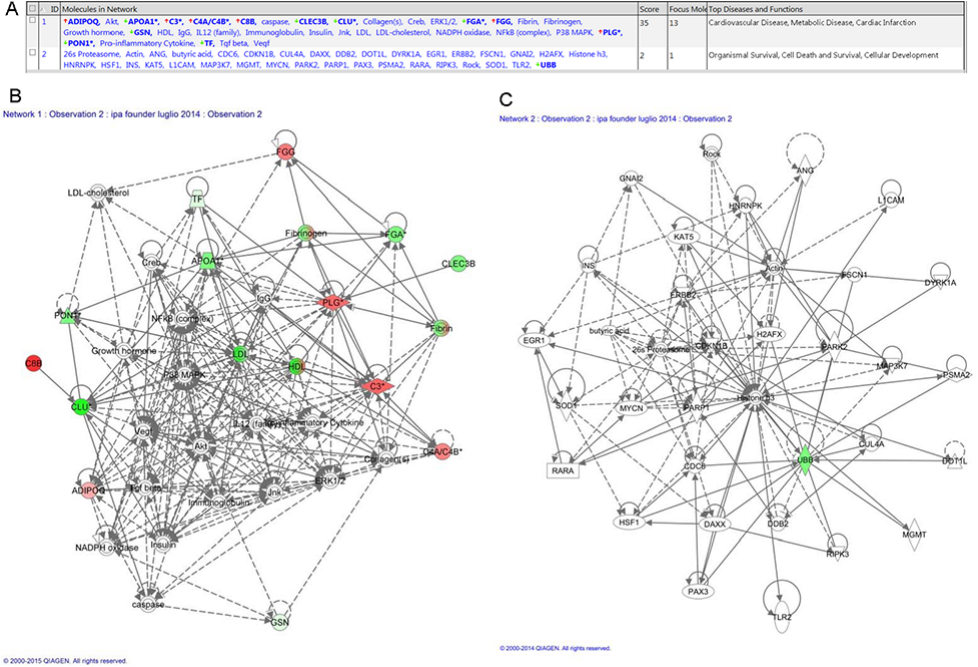
Ingenuity pathway analysis of proteins differentially regulated. (A) List of networks generated from IPA and significantly modulated (log p-value) in healthy carrier vs. controls. Top network (B) 1 and (C) 2.

With regard to the disease analysis, performed on the same set of samples, we found that the plasma proteins were connected to: i) Cancer including abdominal cancer (n molecules = 23, p = 1,65E-07) ii) Cancer including epithelial neoplasia (n molecules = 22, p = 7,35E-06); iii) Cancer including carcinoma (n molecules = 20; p = 2,12E-04), respectively (S4 Table),

IPA analysis was further performed comparing proteins differentially expressed in healthy BRCA1-mutation carriers vs. healthy controls; in this subset, we obtained 2 predicted networks (Fig 4A): i) Cardiovascular Disease, Metabolic Disease, Cardiac Infarction (Fig 4B) and ii) Organismal Survival, Cell Death and Survival, Cellular Development (Fig 4C). Moreover, for links to diseases and bio-functions, we found: Cancer, including abdominal cancer (n molecules = 13, p = 2,74E-04); Cancer epithelial, including neoplasia (n molecules = 13, p = 5,37E-04); Cell Death and Survival, including cell death (n molecules = 12 p = 1,48E-06) (S5 Table).

Finally, IPA software was used to explore potential relations between deregulated proteins and carcinogenesis. This analysis revealed that most of the identified proteins are signaling molecules associated with cell growth, cell death, and cellular metabolism. Among them, gelsolin appeared as the most promising target.

### Western blot of plasma samples

**Fig 5:**
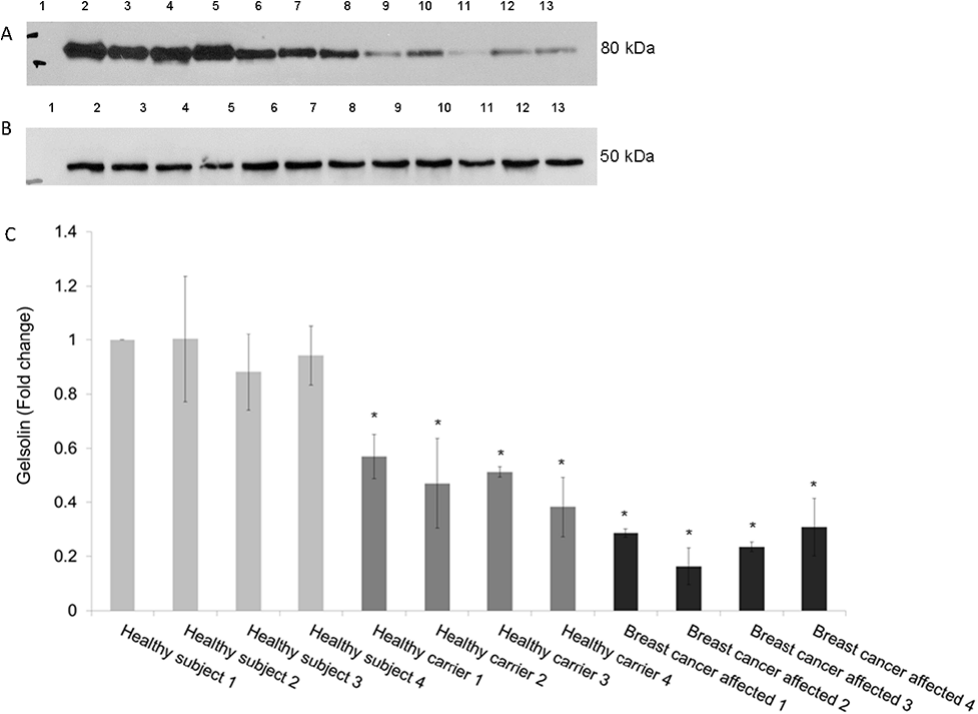
Validation of gelsolin down-regulation by western blot. (A) Analysis was performed on undepleted plasma samples. 50 μg of proteins was loaded in each lane. Lane 1: Molecular weight marker; Lane 2–5: Controls; Lane 6–9: Healthy carrier; Lane 10–13: Cancer patients. (B) ɣ-tubulin blot shows equal amount of protein loading. (C) Densitometric analysis for gelsolin protein levels. Analysis was performed using three independent experiments. Data are mean ± SEM (N = 3). *p < 0.05.

The purpose of this step was twofold: on one hand, to verify that the expression of gelsolin was discriminatory and reproducible among BC patients, healthy carriers and controls, and, on the other hand, to verify if those differences were present in undepleted plasma samples. As shown in Fig 5, the expression of gelsolin was sensibly reduced both in cancer patients and in healthy carriers compared to controls.

### Correlation between gelsolin and BRCA1 expression

To determine whether the expression of gelsolin was associated with the BRCA1 mutation status, we examined gelsolin levels in the following panel of BC cell lines: MCF-7 (sporadic breast ductal carcinoma cell line), HCC1937 (a near tetraploid cell line from breast ductal carcinoma, homozygous for a frameshift mutation in BRCA1), and MCF-7shBRCA1 (MCF-7 cells in which BRCA1 has been transiently silenced by Sh-RNA interference).

**Fig 6:**
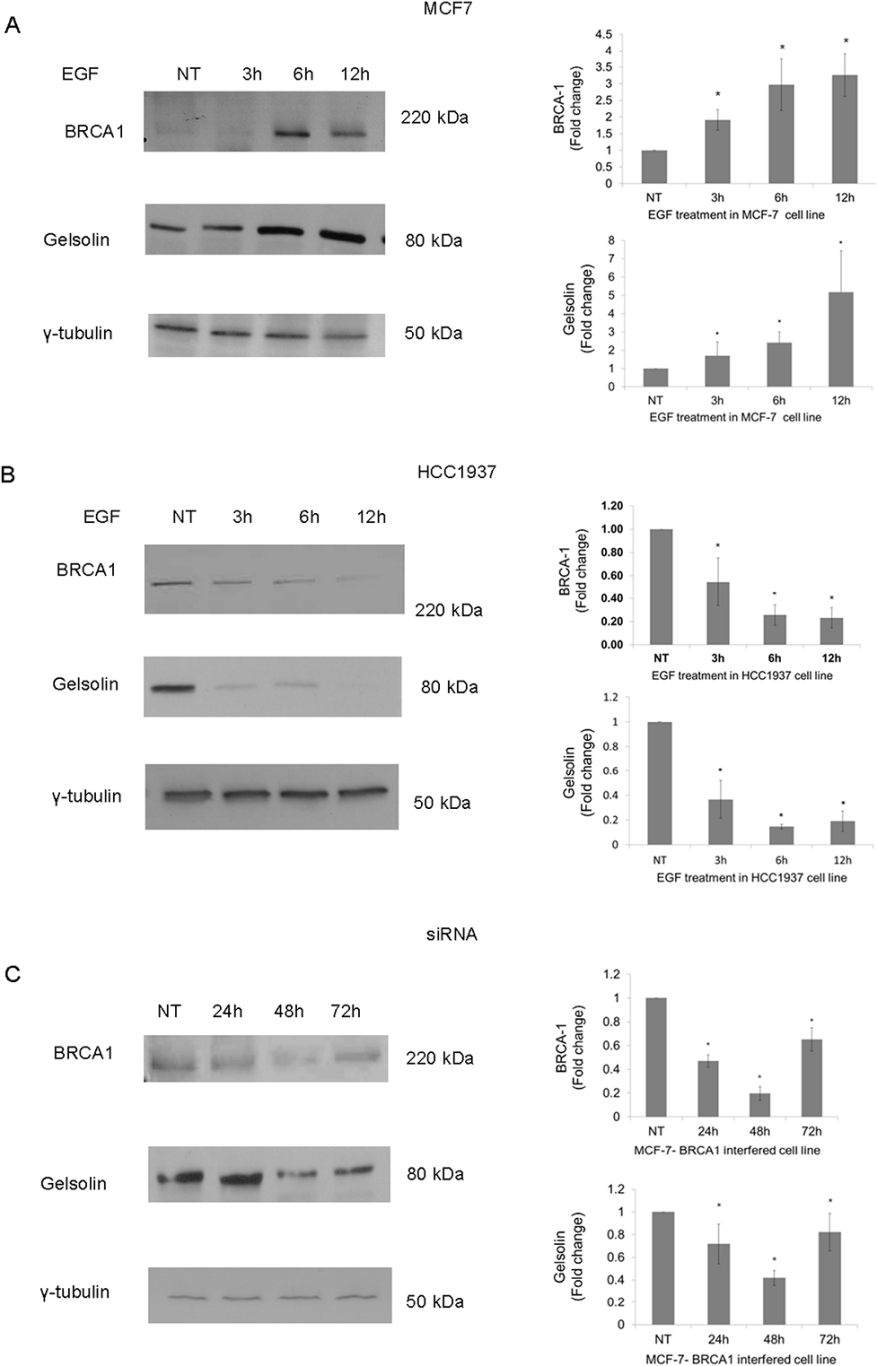
Western blot analysis of BRCA1 protein expression and gelsolin in MCF-7, HCC1937, and MCF-7 ShBRCA1 cells. Analysis was performed on cells extracts. 4–15% precast SDS PAGE (Biorad) was used. 80 μg of proteins was loaded in each lane. BRCA1 and gelsolin expression was assayed in MCF-7 cell line stimulated with EGF at 3, 6 and 12h (A), in HCC1937 cell line stimulated with EGF at 3, 6 and 12h (B) and in MCF-7- BRCA1 interfered cell line. In each cell panel, left panel is representative data of western blot analysis; right panel is showing densitometric analysis for BRCA1 and gelsolin protein levels. Analysis was performed using three independent experiments. Data are mean ± SEM (N = 3). *p< 0.05. In each cell panel ɣ-tubulin blot shows equal amount of protein loading.

In MCF-7 (Fig 6A), Epidermal Growth Factor (EGF) treatment markedly increased the expression of BRCA1, up-regulating gelsolin as well. Conversely, in HCC1937 (Fig 6B), EGF did not affect neither BRCA1 nor gelsolin levels. Lastly, we demonstrated that transient silencing of BRCA1 in MCF-7 cells (Fig 6C) induces down regulation of gelsolin up to 48 h, with a subsequent increase at 72 h, due to the switch-off of interference as previously reported for later time points [32]. These results clearly indicate that gelsolin expression is regulated in a BRCA1-dependent manner.

### Interaction between BRCA1 and ATF1

**Fig 7:**
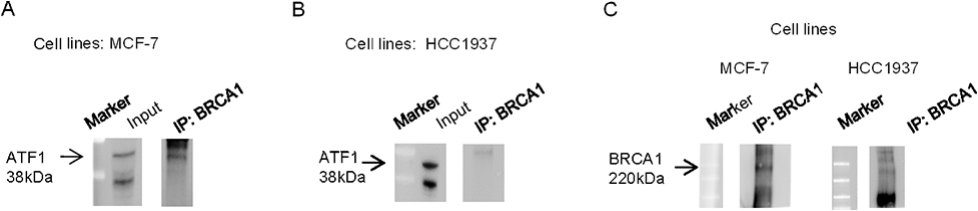
Immunoblotting to demonstrate the association between BRCA1 and ATF1. MCF-7 (panel A) and HCC1937 (panel B) cells were lysed and subjected to immunoprecipitation with the appropriate antibody as described in “Materials and Methods.” The immunoprecipitates were separated by SDS-polyacrylamide gel electrophoresis and transferred to a nitrocellulose membrane. Total cells extract was used as positive control (input). The immunoblot was probed with rabbit anti-ATF1 antisera at 1 mg/ml (C-20 Santa Cruz.) and developed by enhanced chemiluminescence.(C) BRCA1 levels in MCF-7 and HCC1937 human breast cancer cells lines.

Previous studies have demonstrated that BRCA1 can physically interact with the cyclic AMP-dependent transcription factor ATF1 [35]; here, an immunoprecipitation experiment was performed to further assess this interaction. Five hundred μg of proteins extract from MCF-7 and HCC1937 breast cancer cell lines were subjected to immunoprecipitation with a monoclonal antibody directed against the C terminus of BRCA1 (D9-Santa-Cruz), followed by immunoblotting with an anti-ATF1 antibody. In MCF-7, ATF1 was identified by immunoblotting analysis as a 38-kDa species present in BRCA1 immunoprecipitates (Fig 7A, right panel). On the other hand we observed that, in HCC1937, the antibody, directed against BRCA1, was unable of co-precipitating ATF1 (Fig 7B, right panel), probably because the mutation might block or reduce the interaction between BRCA1 and ATF1. It is noteworthy that the amount of ATF1 in whole extract was equivalent in MCF7 and in HCC1937 cells (compare Fig 7A and 7B, left panels): therefore, the increased presence of ATF1 in MCF7-IP is a real experimental finding and not dependent on higher levels of this transcription factor in the cell line. Fig 7C shows the levels of BRCA1 in the two cell lines.

### Role of ATF1 in repression of gelsolin expression

It has recently been shown that ATF1 binds to gelsolin promoter and negatively controls its activity [36]. gelsolin transcription is decreased by ATF1 binding to a 27-bp sequence located approximately 135 bp upstream of the transcription start site. Here, to determine whether the ATF-binding activity on gelsolin promoter was affected by the simultaneous presence of a BRCA1 gene mutation, a chip assay was performed in MCF-7 and HCC1937 cell lines.

To quantify the effects, we carried out a qPCR on the DNA immunoprecipitated by ATF1 antibodies, using a pairs of primers specific for the respective binding sites to the gelsolin promoter, as described in the materials and methods section.

**Fig 8:**
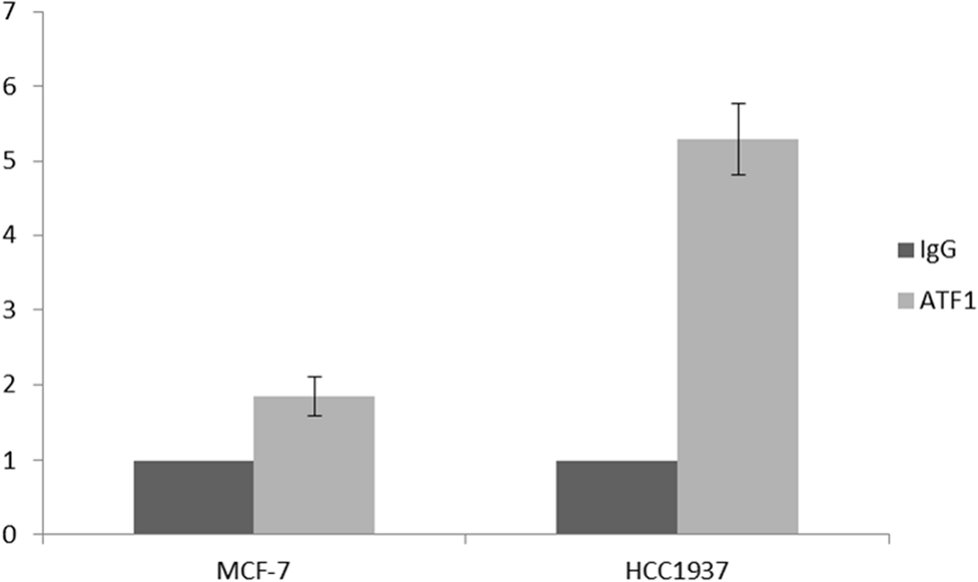
Chip Assay. The binding activity of ATF1 to the gelsolin promoter was evaluated by ChIP analysis. Chromatin was isolated and immunoprecipitated with a specific antibody for ATF1. The binding activity was evaluated by qPCR in MCF7 and HCC1937 human breast cancer cells lines.

Since the reduction of gelsolin levels correlates with the mutation or loss of BRCA1, we hypothesize that BRCA1 regulates the recruitment of ATF1 to the gelsolin promoter. We believe that physical interaction between BRCA1 and ATF1, hinders the binding of the transcription factor to gelsolin promoter; on the other hand, when BRCA1 is absent or mutated, ATF1 is allowed to bind the promoter and to inhibit gelsolin expression. Chip results support this hypothesis, as shown in Fig 8, where the binding of ATF1 to gelsolin promoter is much higher in HCC1937 cell line than in MCF7.

## Discussion

Proteomic-based approaches are increasingly applied to cancer biomarkers discovery. Protein profiles of biological samples determined in a high-throughput fashion allow to identifying proteins that are differentially regulated in cancers and might possibly be novel biomarkers. The blood contains a treasure of biomarkers (many of which are still undiscovered or uncharacterized) that could reflect the ongoing physiologic state of all tissues. Unfortunately, the dynamic range of plasma proteins spans over 10 orders of magnitude, far greater than the measurement capability of current technologies: consequently, potential biomarkers could be entirely masked by the overwhelming abundance of relatively few proteins [37]. In this paper, we show that plasma sample complexity can be effectively reduced with a simultaneous increase in protein identification using a multistep method, confirming that this strategy represents a powerful tool for the identification of specific biomarkers in inherited breast cancer.

Breast cancer has become one of the most intensively studied human malignancies in the post-genomic era; several hundred papers over the last few years have investigated various clinical and biological aspects of human BC using high-throughput molecular profiling techniques.

Given the highly heterogeneous nature of this disease and the lack of robust conventional markers for disease prediction, prognosis, and response to treatment, the enrollment of subjects with a high homogeneous genetic background represents a solid starting point.

BRCA1-derived breast cancers have been shown to leave a characteristic imprinting on the panel of genes expressed by the tumors [38]. Moreover, the occurrence of a BRCA1-like gene expression profile in sporadic BC due to methylation-mediated silencing, highlights the need for the use of subjects with analogous gene expression profiling [39].

Here, proteomic analysis disclosed that gelsolin was down-expressed in plasma samples of patients with hereditary BC, and that its levels were associated with the BRCA1 mutation status, suggesting that this important tumor suppressor gene might promote BC cell proliferation, invasion and migration, also thorough the down-regulation of gelsolin.

The reason for focusing on this specific protein derives from the fact that loss of gelsolin is one of the most frequently occurring molecular defects in BC and it negatively correlates with tumor progression [40–46].

To determine whether the expression levels of gelsolin were associated with the BRCA1 mutation status, we initially examined gelsolin expression in sporadic and BRCA1-mutated BC cell lines; these experiments clearly demonstrated that BRCA1 is indeed directly involved in gelsolin modulation, since no significant changes were detected in sporadic BC cell lines. In the attempt to dissect the molecular mechanisms underlying this phenomenon, we explored the possibility that the transcription factor ATF-1 might be an important player. Dong and coll. [36] have shown that ATF-1 is a strong negative modulator of gelsolin at transcriptional level, through its binding to a 27-bp cis-element mediating sequence located upstream the gelsolin transcription start site. Alongside with it, a robust body of evidence indicates that BRCA1 can physically and functionally interact with ATF-1 [35], enhancing its transcriptional activity. On this basis, we performed functional experiments, confirming that the DNA-binding activity of ATF1 was selectively higher in the BRCA1-mutated cancer cells compared to the sporadic ones and it correlated inversely with the expression of gelsolin, independently on the protein levels of the cAMP response element-binding protein/activating transcription factor.

Therefore, we propose a model in which the tumor suppressor BRCA1 negatively modulates gelsolin expression by physically recruiting ATF-1 on its promoter. The presence of a mutation, which results in an impairment/loss of BRCA1 function, loosens the binding of ATF-1 to the gelsolin promoter, producing a significant increase in its intracellular levels.

Advances in proteomics are contributing to a better understanding of pathophysiology of neoplasia, cancer diagnosis, and anticancer drug discovery. Constant refinement of techniques and methods to determine the abundance and status of proteins hold great promise for the future studies of cancer and the development of innovative cancer therapies.

The plasma-proteomics-based approach proposed in the present study, may provide an answer to this dilemma, i.e. who is going to get cancer and when. Obviously all the findings and conclusions need to be substantiated in a larger cohort, but we are confident that this strategy will trigger more comprehensive studies aimed at improving the potential of early diagnosis of cancer.

## Supporting Information

S1 Fig HPLC depletion chromatograms for each analized plasma samples. (PDF)

S1 Table Densitometric data of 2D gel electrophoresis and protein identification by LC-MS/MS analysis. (XLSX)

S2 Table List of deregulated proteins in breast cancer affected patiens vs healthy subjects with a fold change of at least 1.8, included in IPA analysis. (XLSX)

S3 Table List of deregulated proteins in healthy founder carriers vs healthy subjects with a fold change of at least 1.8, included in IPA analysis. (XLSX)

S4 Table Diseases and bio functions correlated with deregulated proteins in breast cancer affected patiens vs. healthy subjects. (XLS)

S5 Table Diseases and bio functions correlated with deregulated proteins in healthy founder carriers vs. healthy subjects. (XLS)
