# Supplementary material for: Plasma Proteomic Profiling in Hereditary Breast Cancer Reveals a BRCA1-Specific Signature: Diagnostic and Functional Implications
Source: PLoS One. 2015 Jun 10;10(6):e0129762. doi: 10.1371/journal.pone.0129762 (PMC4465499; doi:10.1371/journal.pone.0129762)
Supplement: S1 Fig — (PDF) [file pone.0129762.s001.pdf]

Chromatogram of 4,6x 50 mm column. First panel are relative to representative chromatogram, other panel belong to every run sample. We used for each chromatographic separation 2 mg of plasma samples proteins. This was performed in order to normalize protein concentration for all samples.

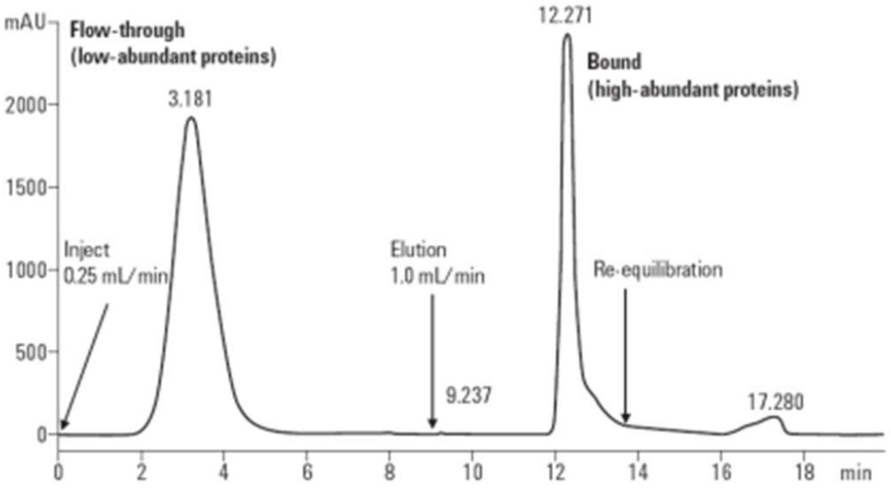

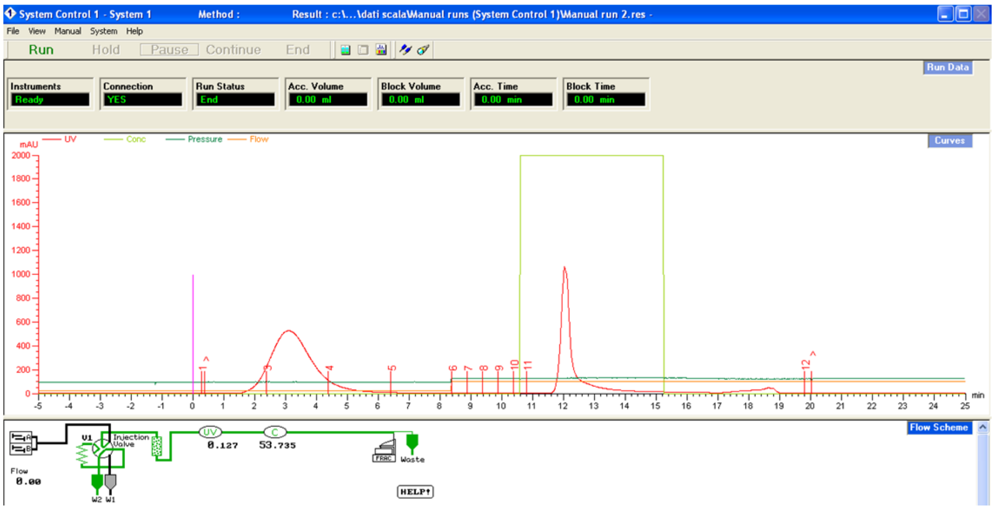

Cancer patient 1

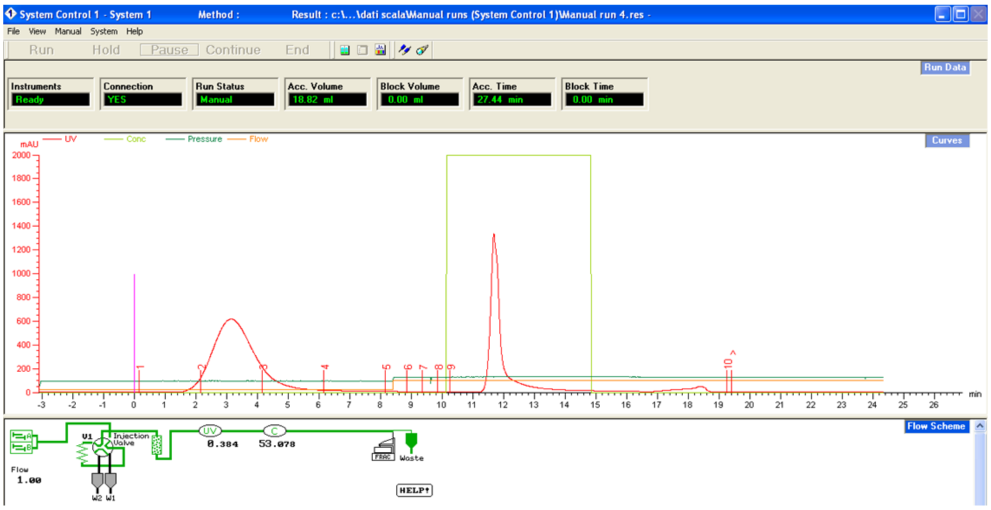

Cancer patient 2

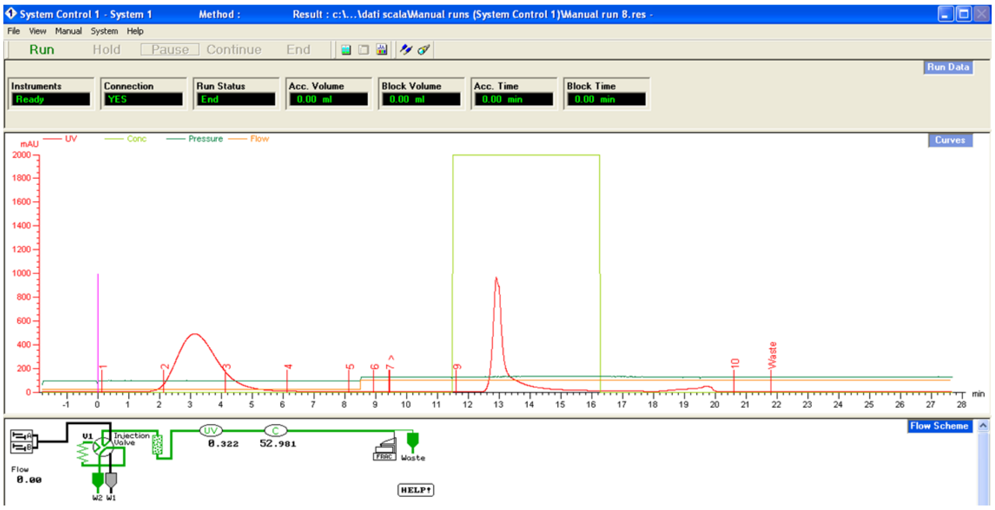

Cancer patient 3

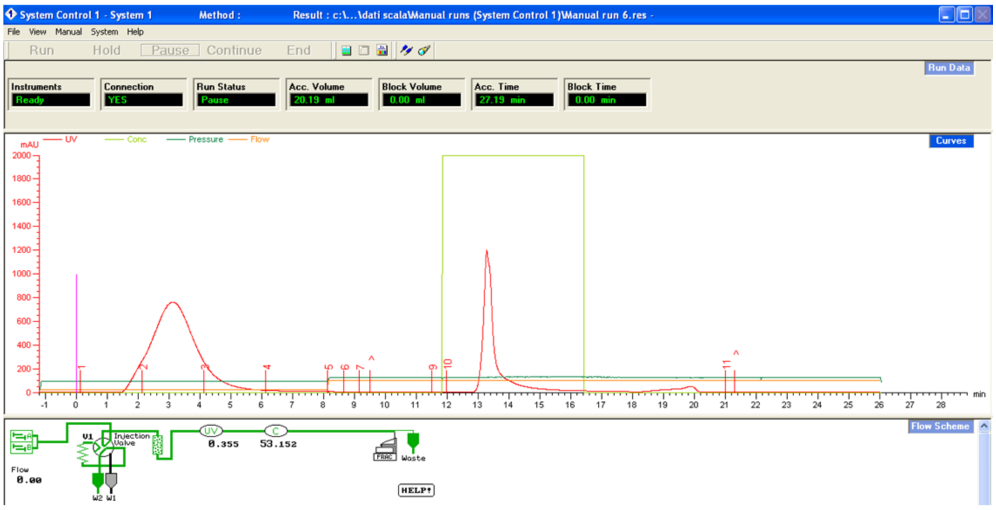

Cancer patient 4

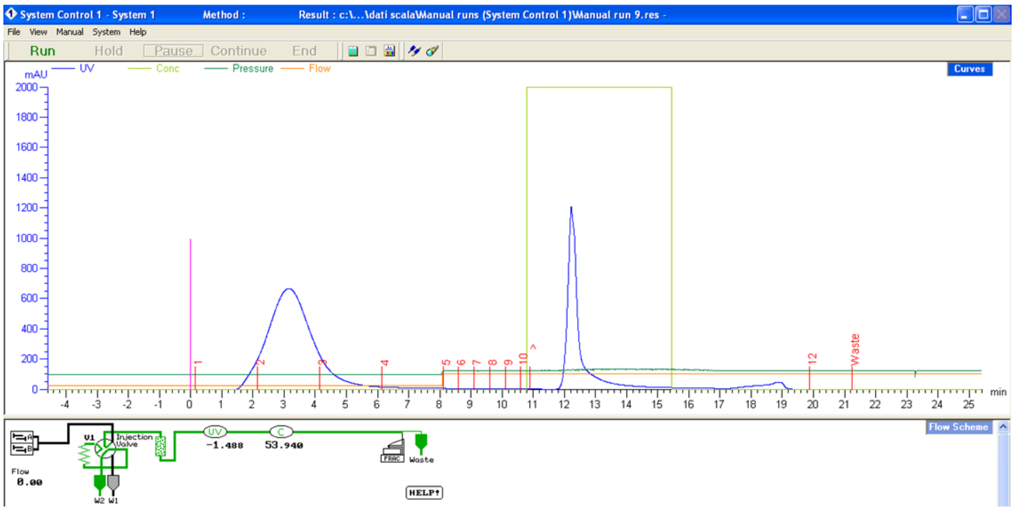

Healthy carrier 1

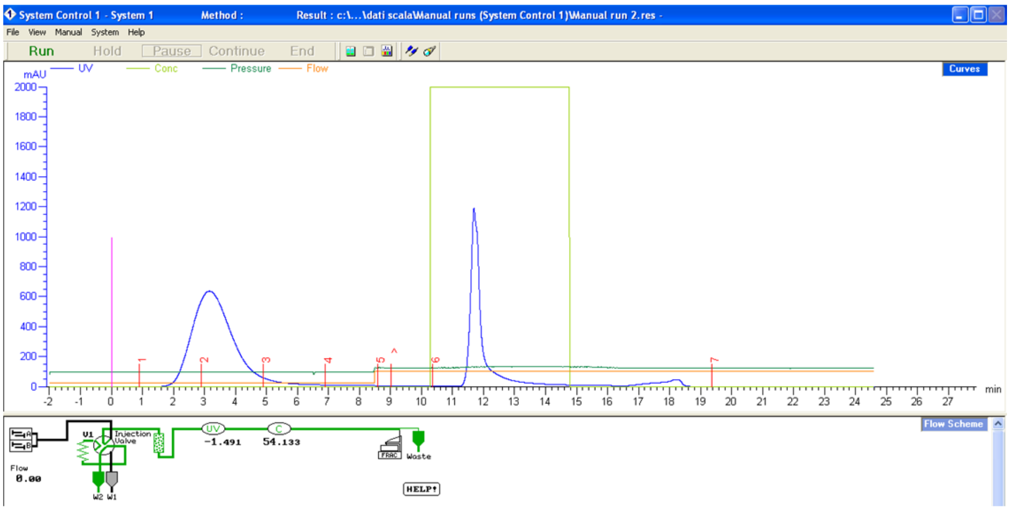

Healthy carrier 2

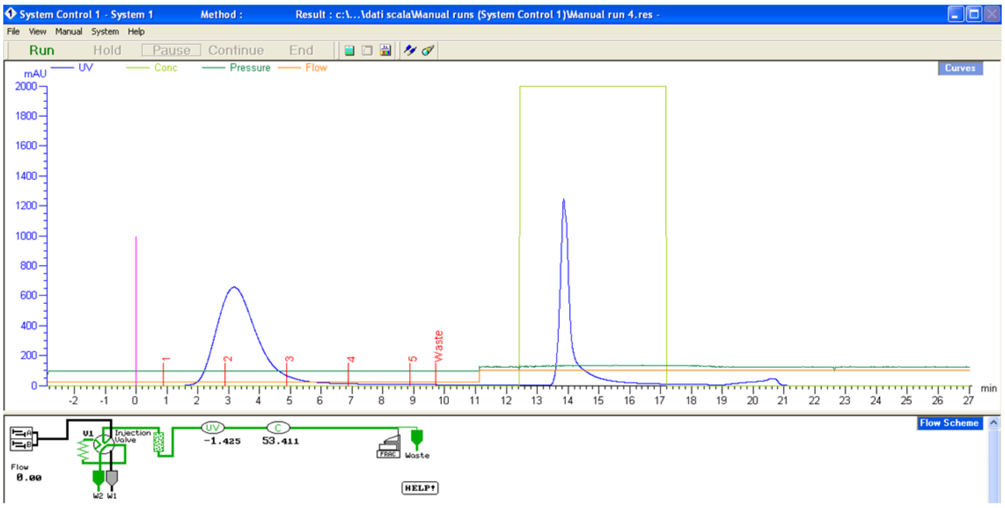

Healthy carrier 3

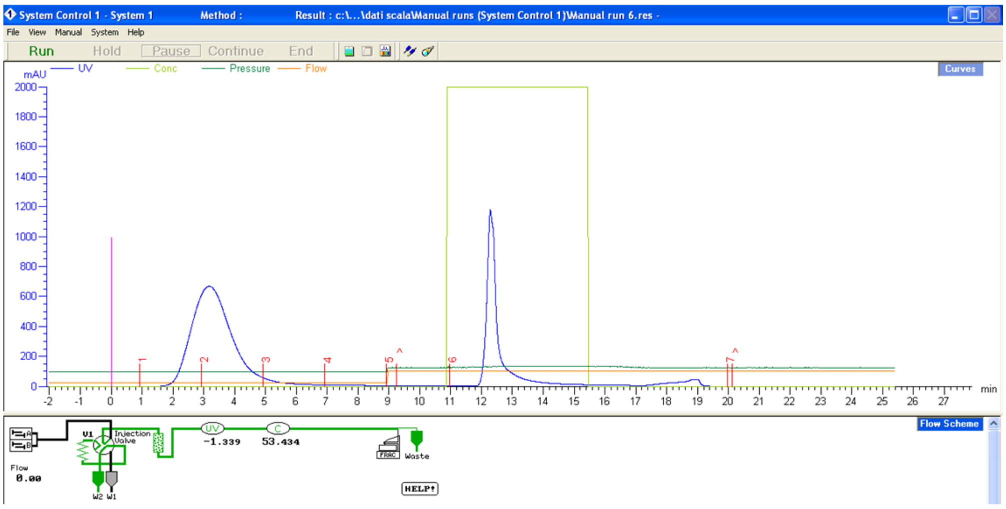

Healthy carrier 4

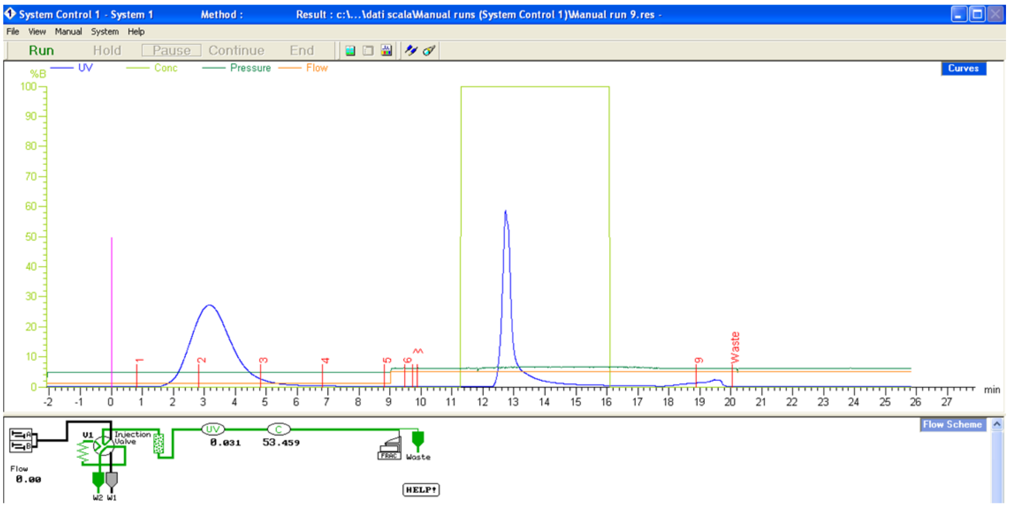

Healthy Control 1

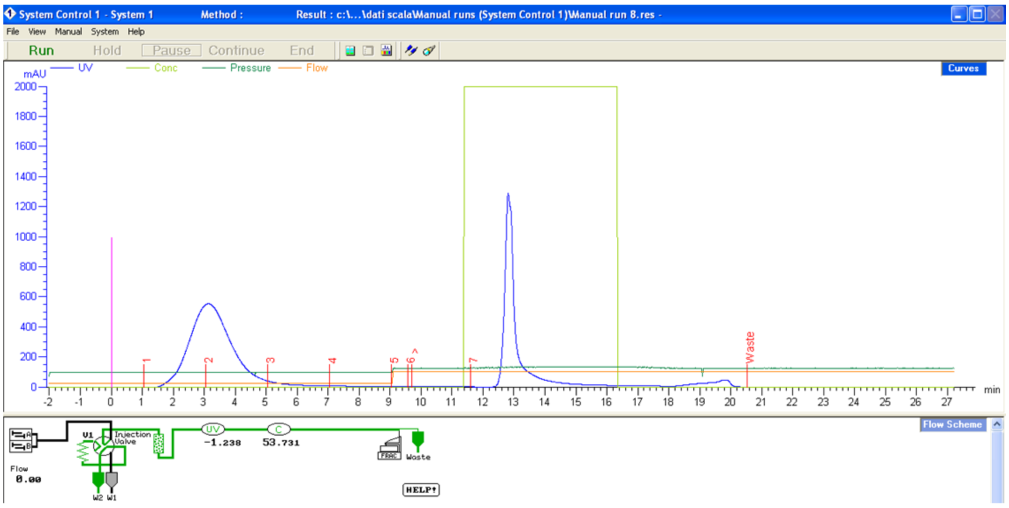

Healthy Control 2

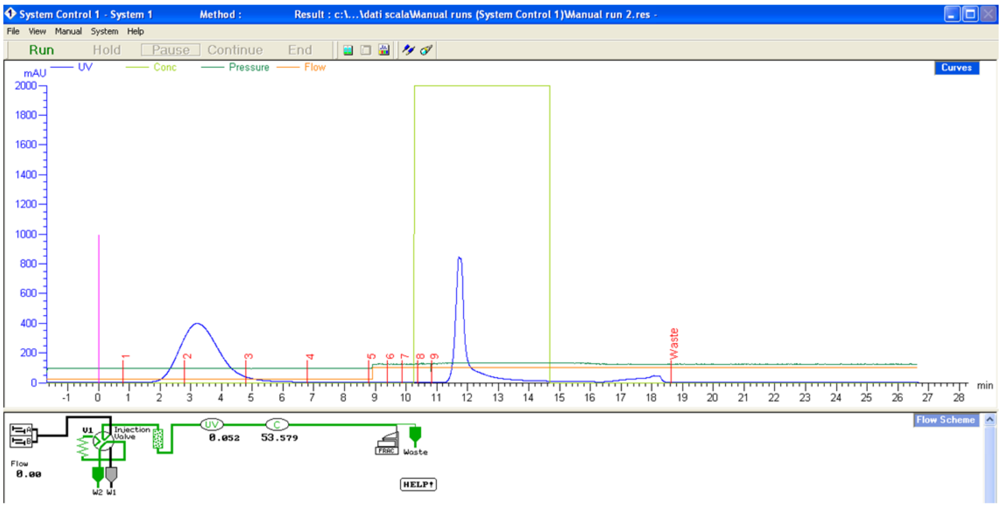

Healthy Control 3

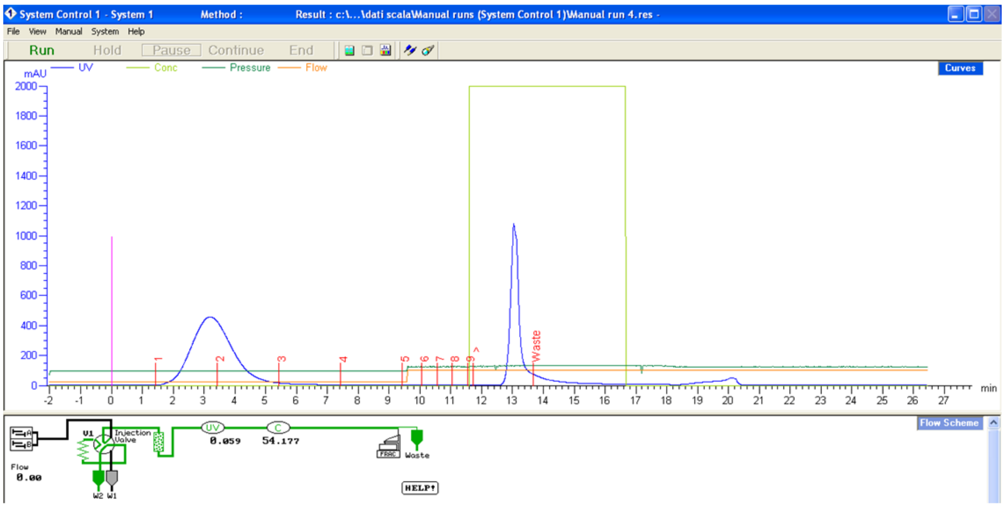

Healthy
Control 4
